# Estimating the number of hand, foot and mouth disease amongst children aged under-five in Beijing during 2012, based on a telephone survey of healthcare seeking behavior

**DOI:** 10.1186/1471-2334-14-437

**Published:** 2014-08-12

**Authors:** Xiaoli Wang, Xiaona Wu, Lei Jia, Xitai Li, Jie Li, Shuang Li, Haikun Qian, Quanyi Wang

**Affiliations:** Beijing Center for Disease Prevention and Control, 16 Hepingli Middle Street, Beijing, China

**Keywords:** Burden, Hand, Foot and mouth disease, Consultation rate

## Abstract

**Background:**

Over the last decade, increases in the number of outbreaks of hand, foot and mouth disease (HFMD) have shifted the disease into the public health spotlight in China. Children under the age of five years are particularly susceptible, with fatalities recorded. However, estimating the burden of HFMD has been difficult to conduct to date.

**Methods:**

In 2012, a cross-sectional survey of healthcare-seeking behaviour for HFMD was undertaken, using computer assisted telephone interviewing (CATI) technology. Sample of telephone numbers was obtained from the Beijing Immunization Information System. Respondents were parents or guardians of children under the age of five. Multiplier model was used to estimate the number of HFMD case, following the telephone survey of healthcare-seeking behavior. The number of laboratory-confirmed cases was also estimated based on the monthly positive rate of each subtype of virus causing HFMD. The age-specific case fatality rate (CFR) was calculated based on the ratio of reported deaths to the estimated number of cases.

**Results:**

For children under five, the consultation rate of parent-defined cases was estimated at 77.8% ((95% CI = [75.2, 80.4]). Parents or legal guardians of children aged between two and four years were more likely to seek healthcare than those of children aged less than two years. For children under the age of five, we estimated that there were 40,165 (95% CI = [38,471, 41,974]) HFMD cases, with an incidence rate of 5.6%, and 22,166 (95% CI = [21,150, 23,295]) laboratory-confirmed cases in Beijing during 2012. The overall CFR was estimated at 10 deaths per 100,000 cases, while for children aged less than two years it was 15.6 deaths per 100,000 cases.

**Conclusions:**

Given the public health impact of HFMD in China, control measures need to be prioritized for children < 2 years, due to the higher CFR in this age group. Sentinel surveillance approaches could be used to monitor trends and the impact of control measures.

**Electronic supplementary material:**

The online version of this article (doi:10.1186/1471-2334-14-437) contains supplementary material, which is available to authorized users.

## Background

Hand, foot, and mouth disease (HFMD) is a common illness caused by viruses that belong to the enterovirus genus (group). Enterovirus 71(EV71) and coxsackievirus A16 (CoxA16) are the major causes for current HFMD cases in China. Enterovirus 71 has long been thought to be associated with severe central nervous system diseases [1, 2]. Children aged less than five years are particularly susceptible to HFMD [3]. Due to the recent increases in outbreaks in China, HFMD is now considered a major public health issue [4].

Since 2007, HFMD surveillance has been performed in Beijing, and relies on measuring consultations for clinically diagnosed cases [4]. The proportion of cases that sought healthcare is critical for determining disease burden. However, there are limited studies examining the healthcare seeking behaviour for HFMD. Due to a lack of systematic assessment of healthcare usage, the true burden of HFMD in Beijing is unknown. Our study aimed to quantify the healthcare seeking behavior of infants and children under the age of five years, and to use this data to estimate the true burden of HFMD in Beijing.

## Methods

### Telephone survey of healthcare-seeking behavior

We conducted a cross-sectional survey of healthcare-seeking behaviour for HFMD between September 25, and November 7, 2012, using computer assisted telephone interviewing (CATI) technology. Parents/guardians of children (born after December 2007) living in Beijing for more than half a year were invited to participate. Potential participants were screened for eligibility at the beginning of each call. All participants provided verbal consent over the telephone prior to the survey interview.

A sample of telephone numbers was obtained from the Beijing Immunization Information System. Children who are administered scheduled vaccines in Beijing are mandated by the Beijing Health Bureau to be enrolled in this system. The following information is uploaded into the registry: name, gender, birth date, and contact phone numbers.

Stratified random sampling was used in this study. Firstly, we divided children into five age groups (0~, 1~, 2~, 3~, and 4 ~ 5). Next, 4,000 contact phone numbers for each age group were randomly selected.

The survey was piloted with 50 parents in order to refine the survey questions. Participants were asked to nominate whether their children had previously had any episodes of rashes on their hands and feet, mouth or buttocks (parent-defined HFMD) during 2012. If an episode was reported, further questions were used to probe whether the child had been attended a health facility and whether their children had been diagnosed with HFMD by a physician. The age, gender and name of hospital/clinic visited were recorded for each child.

Rash was defined as a skin change including a change in the color, appearance or texture. Due to the difficulty for parents/guardians to differentiate HFMD from other diseases that have similar symptoms, the definition of parent-defined HFMD cases was quite broad. The following information was provided to participants to assist them with differentiating HFMD from other diseases:

1. Diaper rash: usually comes on quickly, and affects the parts of the baby’s body that are in close contact with the diaper.
2. Varicella: usually starts with a vesicular skin rash mainly on the body and head rather than on the limbs. The rash then develops into itchy, raw pockmarks, which mostly heal without scarring. It generally occurs during winter and spring.
3. Measles: the rash usually begins several days after the fever starts. It starts on the back of the ears and, after a few hours, spreads to the head and neck before extending to the remainder of the body. The rash is often itchy.
4. Scarlet fever: the rash usually appears firstly on the neck and face (often leaving a clear, unaffected area around the mouth). It then spreads to the chest and back and finally to the rest of the body. The rash may be itchy. Areas of rash usually turn white when pressed. By day six, the rash usually fades, but the affected skin may begin to peel.

### Case numbers estimation

Previously, multiplier models had been successfully used to estimate the burden of pandemic (H1N1) 2009 [5, 6]. In this study, the same approach was used to estimate the burden of HFMD in Beijing, stratified by age. It was assumed that the healthcare seeking behavior for HFMD did not vary by gender and season. The number of HFMD cases was calculated by multiplying the baseline case number *N*_*a*_ by the reciprocal of *C*_*a*_. *N*_*a*_ was the number of probable (clinically diagnosed) cases of HFMD (for age group *a* ) reported in the electronic National Notifiable Infectious Diseases Reporting Information System (NNIDRIS) [7]. *C*_*a*_ was the age-specific proportion of individuals with parent-defined HFMD that sought healthcare (see Equation 1).

1 $$\mathrm{Total}\;\mathrm{symptomatic}\;\mathrm{cases}\;=\;\underset{a}{\sum }\frac{N_{a}}{C_{a}}$$

Due to changes in the positive rate of HFMD over time, the number of laboratory confirmed cases was calculated using: (1) the reported number of probable case of HFMD in NNIDRIS, (2) the positive rate of each subtype (EV71, CoxA16, other subtypes and co-infection), and (3) the proportion of cases that sought healthcare (see Equation 2). Co-infection was defined as a mixed infection with at least two subtypes of virus causing HFMD.

2 $$\begin{array}{c} \mathrm{Total}\;\mathrm{laboratory}‒\mathrm{confirmed}\;\mathrm{cases}\;\\ \;=\;\underset{a}{\sum }\frac{\underset{s}{\sum }\underset{m}{\sum }N_{\text{am}}P_{\text{am}s}}{C_{a}} \end{array}$$

*N*_*am*_ was the number of HFMD consultations (in month *m* and for age group *a*) reported in NNIDRIS. *P*_*ams*_ was the monthly positive rate of each subtype for age group *a. C*_*a*_ was the age-specific proportion of individuals with HFMD that sought healthcare. Age-specific case fatality rate (CFR) was calculated from the ratio of reported HFMD deaths to the estimated number of cases.

In NNIDRIS, a probable case of HFMD was defined as a patient with a papular or vesicular rash on the hands, feet, mouth, or buttocks, with or without fever. A confirmed case was defined as a probable case with laboratory evidence of enterovirus infection (including EV71, CoxA16, or other non- EV71 and non-CoxA16) detected by RT-PCR, real-time PCR, or virus isolation. Probable and laboratory confirmed cases were reported online in NNIDRIS within 24 hours of diagnosis, via the use of a standardized form. The following information was captured in the system: basic demographic information (gender, date of birth, and address), severity (mild or severe), death status, date of symptoms onset, date of diagnosis, and date of death (if applicable). Clinical samples, including throat swab, rectal swab, faecal sample, vesicular fluid, or cerebrospinal fluid, were collected from the first five HFMD cases who visited a hospital outpatient department and who had mild, probable disease. In addition, swabs were collected from all severe cases and from all children with HFMD who died [8]. EV71 and CoxA16 were routinely detected. The China CDC recommended a commercial licensed kit for the detection of EV71 and CoxA16. The detection method was based on a one-step real-time RT–PCR assay.

### Data analysis

95% confidence intervals (CIs) for parent-defined HFMD cases seeking healthcare, stratified by age and gender were calculated using the normal approximation. 95% CIs of estimated cases and estimated rates were determined by Monte Carlo simulation, using a multiplier model (Impact2009, version 1.0) developed by United States Centers for Disease Control and Prevention (CDC) (5). Differences between groups were tested for significance using chi-square test. Data analyses were performed using SPSS statistical software package version 19.0 (IBM SPSS Inc., Chicago, IL, USA). All statistical tests were 2-sided, and significance was defined as *p* < 0.05.

This study was approved by the Human Research Ethics Committee of Beijing Center for Disease Prevention and Control.

## Results

### Healthcare seeking behaviors

During the study period, 20,000 phone calls were made and contact was established with 12,856 (64.3%). No statistically significant differences were found in the distribution by age (*p* = 0.147) and gender (*p* = 0.161) between the children of contacted and those of non-contacted subjects. Of the 12,856 contacted numbers, 11,971 (93.1%) were eligible for the survey, and 6,209 (52%) agreed to participate in the telephone survey. There were no significant difference in terms of age (*p* = 0.206) and gender (*p* = 0.072) between children of non-participants and those of participants. Of the children whose parents participated in our survey, 53.2% were male, 47.6% were under the age of 2 years, most of who were under kindergarten age (2–6 years of age).

As shown in Table 1, a total of 964 parents/legal guardians reported that their child had previously had an episode of syndrome clinically compatible with HFMD, of which 746 was assessed by a physician (77.4%, CI 95% = [74.7, 80.0]). Only 162/746 cases were diagnosed as probable HFMD cases by a physician. There were no significant differences between boys and girls in the occurrence of both of parent-defined or clinically diagnosed HFMD. However, the incidence rate of parent-defined HFMD and clinically diagnosed HFMD among children aged <2 years was significantly different from children aged between two and four years (*p* < 0.001). In addition, for children aged <2 years, only 9.1% of parent-defined cases were finally diagnosed as probable cases by physicians, in contrast to 40.3% of parent-defined cases in children aged between two and four years.

**Table 1:** **Demographic characteristic of study subjects and general results of telephone survey**

Age group (y)	No. subjects		No. parent-defined cases		No. consultations		No. probable cases	
	Male	Female	Male	Female	Male	Female	Male	Female
0-1	1572	1385	308	288	234	214	25	16
2-4	1734	1518	187	181	153	145	56	65
Total	3306	2903	495	469	387	359	81	81

Table 2 showed the consultation rates of parent-defined HFMD cases. Parents of children between the age of two and four were more likely to seek healthcare than those of children aged <2 years (81%% vs.75.2%, *p* = 0.036). However, there was no significant difference in the consultation rate observed between boys and girls (78.2% vs. 76.5%, *p* = 0.544).

**Table 2:** **Consultation rates in parent-defined HFMD cases, Beijing, China**

Characteristics	No. consultation	Consultation rate,%	95% CI	
Gender				
Male (n = 495)	387	78.2	74.5	81.8
Female (n = 469)	359	76.5	72.7	80.4
Age group, y				
0-1 (n = 596)	448	75.2	71.7	78.6
2-4 (n = 368)	298	81.0	77.0	85.0

### Estimates of case numbers

During 2012, a total of 38,528 HFMD consultations and four deaths were reported in Beijing, 82.6% (31,842/38,528) of which were children aged <5 years. 9,668 (30.4%) HFMD cases were aged <2 years and 22,174 (69.6%) were between the age of two and four.

**Figure 1:**
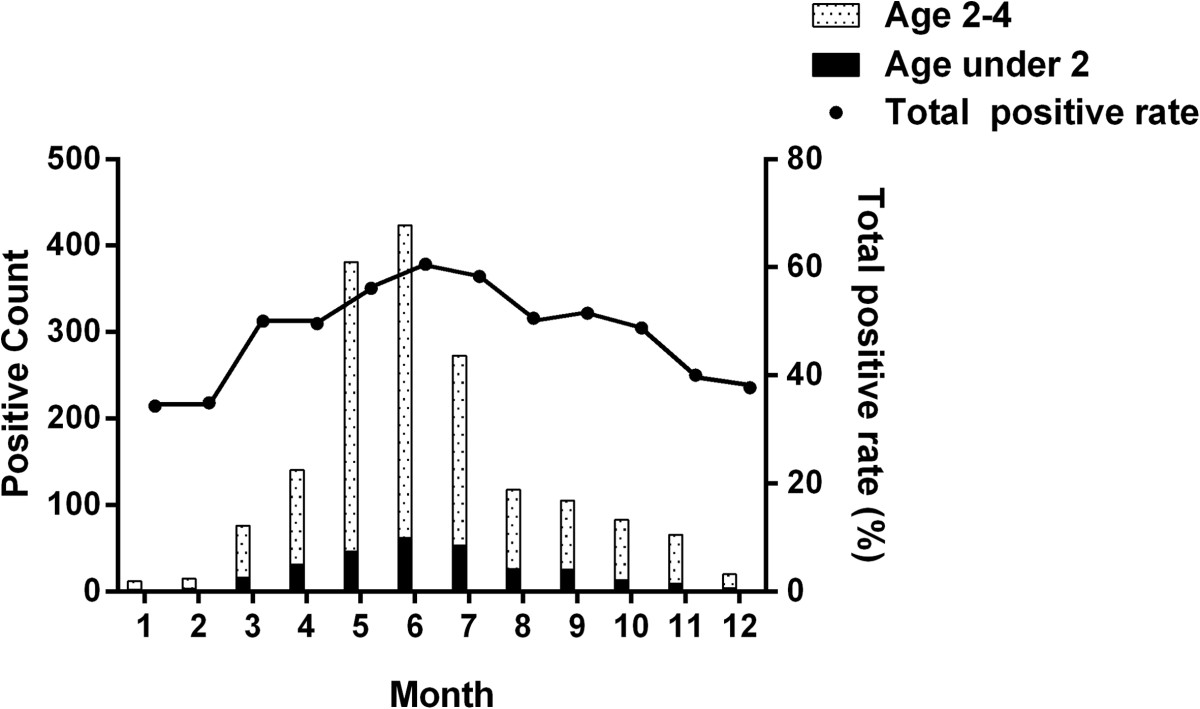
Monthly isolates and total positive rate of virus causing HFMD during 2012, Beijing, China*. *During 2012, a total of 3,186 samples were collected from the first five patients with mild, probable disease who visited hospital outpatient departments every week in each of the 16 districts in Beijing, and from all severe cases and deaths. 1,714 (53.8%) isolates were positive. For children aged less than two years, 52.7% (290/550) of the collected samples were positive. For those aged between two and four years, the positive rate was relatively higher, being 54.0% (1,424/2,636).

Using the multiplier model and the age-specific consultation rate, we estimated that there were 40,165 (95% CI = [38,471, 41,974]) HFMD cases amongst children aged <5 years in Beijing during 2012, with an incidence rate of 5.6 cases per 100 children, as shown in Table 3. Children between the age of two and four had a higher risk for HFMD than those aged <2 years (6.2% vs. 4.7%, *p* < 0.05).Virological surveillance data for HFMD showed that the total positive rate of virus causing HFMD changed significantly from month to month (Figure 1). The overall positive rate increased from January (34.3%) to June (60.6%) and then decreased gradually. There was single peak in the whole year.

**Table 3:** **Estimated numbers of probable cases of HFMD and incidence rates, by age group, Beijing, China**

Age group, y	Estimated no. probable cases, mean (95% CI)	Estimated rate, %, mean (95% CI)
0-1	12,805 (12,280-13,324)	4.7 (4.5-4.9)
2-4	27,360 (26,191-28,650)	6.2 (5.9-6.5)
Total	40,165 (38,471-41,974)	5.6 (5.4-5.9)

We also estimated that a total of 22,166 (95% CI = [21,150, 23,295] HFMD cases were laboratory confirmed in Beijing in 2012, with an incidence rate of 3.1 cases per 100 children under five years, shown in Table 4.

**Table 4:** **Estimated numbers of laboratory confirmed cases of HFMD and incidence rates, by age group, Beijing, China**

Age group, y	Estimated no. lab confirmed case, mean (95% CI)	Estimated rate,%, mean (95% CI)
0-1	7,088 (6,782-7,434)	2.6 (2.5-2.7)
2-4	15,078 (14,368-15,861)	3.4 (3.2-3.6)
Total	22,166 (21,150-23,295)	3.1 (3.0-3.3)

For children aged <2 years, 7,088 laboratory confirmed cases were estimated (95% CI = [6,782, 7,434]. CoxA16 was the predominant circulating virus strain, accounting for 48.6%, followed by EV71 (34.5%) and other subtypes (15.9%). Co-infection was reported in only 1.0%. Similarly, for children between two and four years of age, there was an estimated 15,078 laboratory confirmed cases (95% CI = [14,368, 15,861]. CoxA16 was again the predominant circulating virus strain, accounting for 53.9 percent, followed by EV71 (34.8%), with co-infections reported in 1.8%.

A total of four HFMD deaths were reported (two deaths for each age group). The total CFR was estimated at 10 deaths per 100,000 cases (95% CI = [9.5, 10.4]). The CFR in children <2 years of age was higher at 15.6 deaths per 100,000 (95% CI = [15.0, 16.3]). Whereas, the CFR for children aged between two and four years was 7.3 death per 100,000 (95% CI = [7.0, 7.6]).

### Impact of recall period

Considering the recall bias, we calculated the consultation rate of self-defined HFMD cases with a recall period of 2 weeks. The consultation rate was estimated at 78.4% (95% CI = [71.2%, 85.8%]). No significant difference in the consultation rate was observed between two recall periods (two weeks vs. eleven months).

## Discussion

From the available data, it was estimated that 40,165 probable HFMD cases occurred in children aged less than five years from Beijing during 2012, with an incidence rate of 5.6%, which was higher than the overall level of China [8]. CFR in children aged under two years was higher with 15.6 deaths per 100,000. Furthermore, a total of 22,105 HFMD cases were laboratory confirmed, of which nearly 35% were infected with EV71. EV71 has long been regarded as the predominant strain responsible for severe cases, outbreaks and even deaths. Therefore, the burden of HFMD has been significant in Beijing and has been posing great threats to health and safety of children under the age of five years.

Previously, attempts had been made to estimate the disease burden for pandemic influenza H1N1 2009 and seasonal influenza based on healthcare seeking behavior surveys [9, 10]. However, there have been very few studies conducted for HFMD. This is the first time a telephone survey of healthcare seeking behavior has been conducted to estimate the true burden of HFMD cases in Beijing, China. Our results showed that about 78% of parent-defined HFMD cases would consult a physician, which is higher than the consultation rate for influenza [9–11]. This is perhaps not surprising, when you consider that the most susceptible population for HFMD is children aged less than five years.

We observed that children between the age of two and four had a higher risk for HFMD than those less than two years. In China, most children start kindergarten at the age of two to three years. For children younger than two years, they are often cared for in their home by their paternal grandparents or parents. Attending kindergarten has often been considered as a risk factor for HFMD virus infection, especially for EV71, given the increased person-to-person contact that occurs in the setting [12, 13]. In China, kindergartens and childcare centers are encouraged to engage and educate parents around issues such as HFMD. It is perhaps not surprising that we found that there was a better understanding of HFMD amongst parents/guardians of children aged older than two years. Hence, the parent-definition of HFMD cases for kindergarten age children seemed to better match with the clinical definition.

Our study has several potential limitations. Theoretically, the number of probable HFMD case should be estimated based on the proportion of probable HFMD cases seeking health care. However, it was not feasible to obtain the consultation rate of probable HFMD cases as parents/guardians were unable to determine whether their children were probable cases or not. In this study, instead of using the consultation rate of probable cases of HFMD, we used the consultation rate of parent-defined HFMD cases to adjust for the number of probable cases of HFMD. The definition of parent-defined HFMD cases was so broad that such diseases as varicella or measles may have been included. As our results showed, a proportion of the parent-defined HFMD cases were not clinically diagnosed as HFMD, particularly in the younger children. This probably reflects the higher prevalence of rash-inducing infections occurring in children under 2 years, such as varicella and measles. In this study, we assumed that amongst the parent-defined HFMD cases that did not consult a physician, the ratio of probable HFMD cases and other disease cases was the same as those amongst the cases of parent-defined HFMD that did consult a physician. However, given the increased attention on HFMD in China, the proportion of probable HFMD cases among those parent-defined cases who did not consult any physicians may be smaller. Therefore, the estimated consultation rate for HFMD in our study might have been underestimated. Similar to other retrospective telephone surveys, the refusal of households to respond and the impact of recall bias may have the potential to limit our findings. As shown in the results, recall bias seems to be limited since the estimated consultation rate using a two-week recall period was not significantly different. Additionally, we assumed that there was no change in the consultation rate over time, which may limit the generalization of our findings. In support of the findings in this paper, are the results from a small cross-sectional survey of HFMD amongst children under five that was conducted in August, 2013 (unpublished). The consultation rate of parent-defined HFMD cases from this comparator survey was estimated at 75.1% (95% CI = [66.4%, 83.8%]), which is not significantly different from our results. The change in consultation rate for HFMD over time seems to be limited.

## Conclusion

The burden of HFMD has been significant in Beijing of China, and has been posing a threat to the health and safety of children under the age of five years. Given the public health impact of HFMD in China, control measures need to be prioritized for children < 2 years, due to the higher CFR in this age group. Better surveillance, as well as frequent formal and informal communication and interaction with parents could raise their awareness and facilitate the prevention and control of HFMD.

## Authors’ original submitted files for images

Below are the links to the authors’ original submitted files for images. Authors’ original file for figure 1

## Abbreviations

(HFMD): Hand, foot and mouth disease
(CFR): Case fatality rate
(EV71): Enterovirus 71
(CoxA16): Coxsackievirus A16
(CATI): Computer assisted telephone interviewing
(CDC): (NNIDRIS): National notifiable infectious diseases reporting information system
(CI): Confidential interval.
