# Supplementary figures and images for: Estimating the number of hand, foot and mouth disease amongst children aged under-five in Beijing during 2012, based on a telephone survey of healthcare seeking behavior
Source: BMC Infect Dis. 2014 Aug 12;14:437. doi: 10.1186/1471-2334-14-437 (PMC4149051; doi:10.1186/1471-2334-14-437)

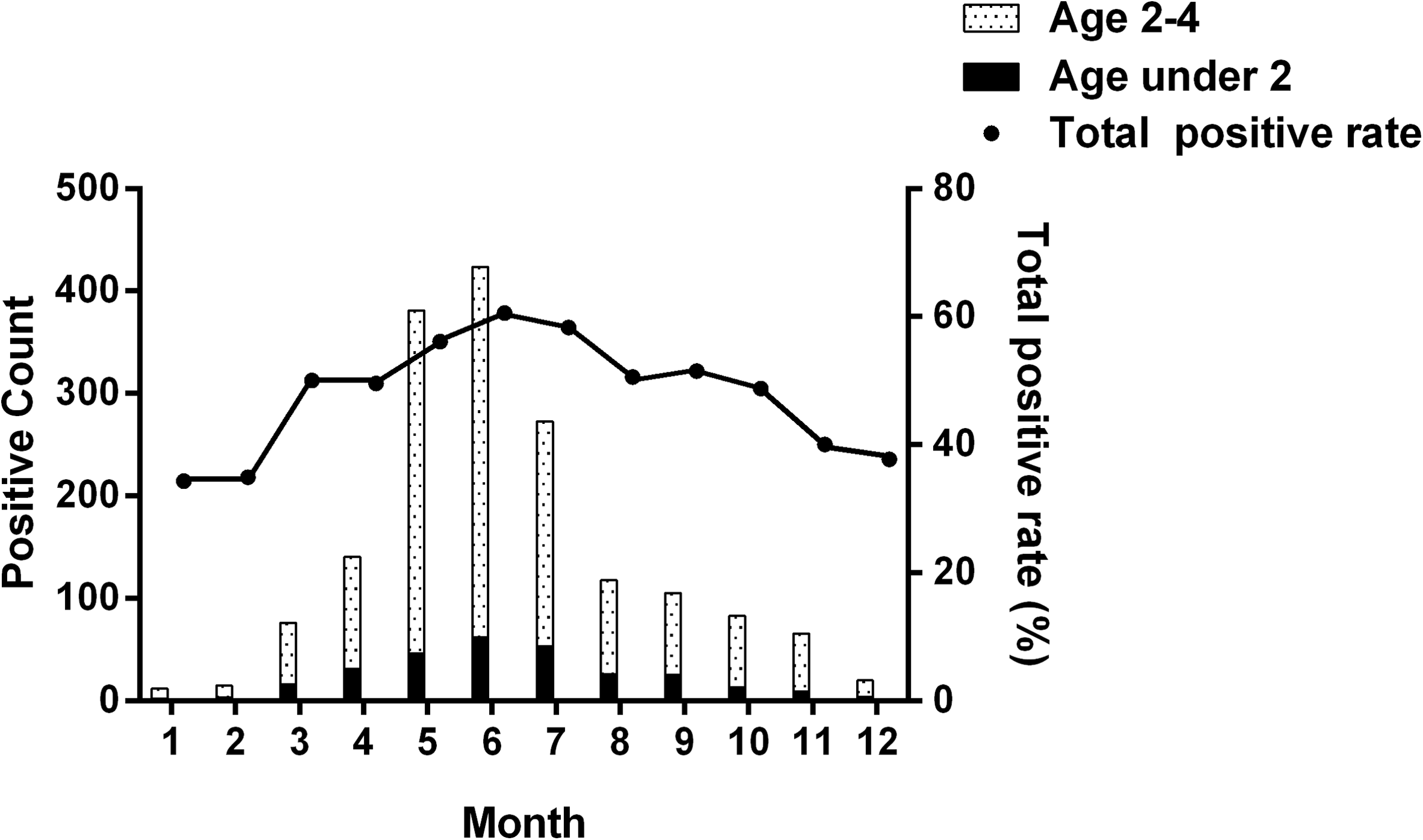

Supplement: Supplementary file 1 — Authors’ original file for figure 1 [file 12879_2013_3748_MOESM1_ESM.tif]
